# Congenital Cytomegalovirus Mortality in the United States, 1990–2006

**DOI:** 10.1371/journal.pntd.0001140

**Published:** 2011-04-26

**Authors:** Benjamin N. Bristow, Kaitlin A. O'Keefe, Shira C. Shafir, Frank J. Sorvillo

**Affiliations:** 1 Department of Epidemiology, School of Public Health, University of California Los Angeles, Los Angeles, California, United States of America; 2 Los Angeles County Department of Public Health, Los Angeles, California, United States of America; 3 Trident University International, Cypress, California, United States of America; Virginia Commonwealth University, United States of America

## Abstract

**Background:**

Congenital cytomegalovirus (CMV) infection is the most common intrauterine infection in the United States disproportionately affecting minority races and those of lower socio-economic class. Despite its importance there is little information on the burden of congenital CMV-related mortality in the US. To measure congenital CMV-associated mortality in the US and assess possible racial/ethnic disparities, we reviewed national death certificate data for a 17-year period.

**Methods:**

Congenital CMV-associated deaths from 1990 through 2006 were identified from multiple-cause-coded death records and were combined with US census data to calculate mortality rates.

**Results:**

A total of 777 congenital CMV-associated deaths occurred over the 17-year study period resulting in 56,355 years of age-adjusted years of potential life lost. 71.7% (557) of congenital CMV-associated deaths occurred in infants (age less than 1 year). Age-adjusted mortality rates stratified by race/ethnicity revealed mortality disparities. Age-adjusted rate ratios were calculated for each racial/ethnic group using whites as the reference. Native Americans and African Americans were 2.34 (95% CI, 2.11–2.59) and 1.89 (95% CI, 1.70–2.11) times respectively, more likely to die from congenital CMV than whites. Asians and Hispanics were 0.54 (95% CI, 0.44–0.66) and 0.96 (95% CI, 0.83–1.10) times respectively, less likely to die from congenital CMV than whites.

**Conclusions/Significance:**

Congenital CMV infection causes appreciable mortality in the US exacting a particular burden among African Americans and Native Americans. Enhanced surveillance and increased screening are necessary to better understand the epidemiology of congenital CMV infection in addition to acceleration of vaccine development efforts.

## Author Summary

Cytomegalovirus (CMV) is a member of the herpes family of viruses, which is transmitted by sexual and non-sexual contact. Human CMV causes a wide variety of infection and illness in healthy adults, in those with compromised immune systems (such as AIDS), in those with cardiovascular disease, and in pregnant women who can pass the infection to their unborn child (congenital CMV). Treatment options for congenital CMV are limited and no effective vaccine to protect against CMV currently exists. Previous studies have demonstrated that African Americans and Mexican Americans are at an increased risk for congenital CMV infections. In this study, the authors examined death certificate data of US Residents from 1990–2006 in which congenital CMV was listed as one of the diagnoses at death. The analysis demonstrated that there is a significant burden of congenital CMV deaths in infants (<1 year old) with African Americans and Native Americans overrepresented. This study helps quantify congenital CMV deaths among US residents and adds further support to the importance of funding CMV vaccine research.

## Introduction

Cytomegalovirus (CMV) is a member of the herpesviridae family of viruses, which cause latent and reactivated viral infection. Humans are the main reservoir of human CMV (human herpes virus 5) [1], [2]. CMV is shed in saliva, urine, breast milk, semen and blood and is spread via direct contact (non-sexual and sexual) [3]. CMV can be vertically transmitted from a mother with primary or secondary infection to her unborn child with consequences ranging from asymptomatic infection to lifelong disability to severe disease resulting in fetal or neonatal death. Secondary infection may be the result of reactivation from a primary infection or new infection. While the majority of cases of symptomatic congenital disease occur following primary maternal infection, secondary recurrent maternal infection can also lead to disease [2], [4].

One of the most important risk factors for primary CMV infection during pregnancy is prolonged exposure to young children since CMV infected children under age two will secrete virus in the saliva and urine for an average of 24 months [5], [6]. This may explain the risk of primary CMV infection during pregnancy in multiparious women, especially in women with children in day care as well as the increased risk in women of childbearing ages working in day care facilities [3], [7].

Sexual contact is another risk factor for maternal infection with vertical transmission. Young maternal age, greater numbers of sexual partners, abnormal cervical cytology, and having a sexually transmitted infection during pregnancy are all associated with CMV seropositivity [3], [8]–[10]. There is limited evidence suggesting presence of a sexually transmitted infection, including HIV, may increase the risk of vertical CMV transmission from mother to fetus/infant [11]–[14].

Congenital CMV infection is the most common intrauterine infection in the United States with direct annual costs of over one billion dollars [15], [16]. The live birth prevalence of congenital CMV infection in the developed world is 0.6–0.7% [12]. Of those infected, 10% are symptomatic as neonates (<30 days of age) with the majority surviving the initial infection; however, greater than 90% develop long-term neurological sequelae including sensorineural hearing loss (unilateral and bilateral), mental retardation, cerebral palsy, and impaired vision from chorioretinitis [4], [9], [17]. Of the remaining 90% of congenital CMV infections that are asymptomatic, approximately 10–15% will later develop long-term neurological sequelae [9], [17]. Congenital CMV infection leads to an estimated 8000 cases of permanent neurologic disability annually [15]. There are more cases of permanent disability due to congenital CMV than other, better known, congenital conditions such as Downs syndrome, fetal alcohol syndrome and spina bifida [11], [18].

Congenital CMV has been labeled as one of the *Neglected Infection of Poverty* in the United States as it is poorly known by the US public-health community, it has a disproportionate impact on the health of the poorest Americans, and it promotes poverty via its negative impact on child development and pregnancy outcomes [19], [20].

Despite its importance, there is a lack of information about congenital CMV-related deaths in the US. Information on congenital CMV mortality is important to better understand the burden of disease and evaluate the effectiveness of public health interventions. Population-based mortality data has been used to investigate other infectious diseases, but these data have not yet been used in published congenital CMV research [21]–[24]. We examined national mortality data to assess the burden and demographics of congenital CMV-associated mortality in the United States from 1990–2006.

## Methods

Multiple-cause-of-death data from US death certificates from the National Center for Health Statistics (NCHS) were analyzed for the years 1990–2006 [25]. These death certificates contain basic demographic information for each decedent, including age, sex, race/ethnicity and state of residence. In addition to designating underlying causes, the physician or coroner completing the death certificate may list up to 20 conditions that are believed to have contributed in some way to the death of an individual. Each of these conditions is coded on the basis of the *International Classification of Diseases* (ICD) system for the year in which the death occurred (*ICD*, *Ninth Revision* [ICD-9] for the period 1990–1998 and *ICD*, *Tenth Revision* [ICD-10] for the period 1999–2006).

We defined cases as US resident deaths having an ICD-9 code of 771.1 (“Congenital cytomegalovirus infection, Congenital cytomegalic inclusion disease”) or an ICD-10 code of P35.1 (“Congenital cytomegalovirus infection”) listed as an underlying or contributing cause on the death record.

Mortality rates were calculated using bridged-race population estimates derived from US census data and were subsequently age-adjusted with weights from the 2000 US standard population data [26]–[28]. Mortality rates for race/ethnicity, sex, age, year and State were calculated with aggregated data from all years of our study to ensure stable rates. Race/ethnicity was divided into 5 categories: non-Hispanic white, non-Hispanic black, Hispanic, Asian/Pacific Islander, and Native American. Analysis by age included infants (<1 year), neonates (<28 days), postneonates (28–364 days), ≤5years and >5 years. For our geographic analysis, we divided the 50 states into 4 major regions according to US census divisions [29]. All calculations were performed with SAS, version 9.1.

## Results

We identified 777 congenital CMV-associated deaths during the period 1990–2006, representing 0.002% of the 39,755,734 total deaths among US residents. 43.8% (340) of deaths were in males and 56.2% (437) were in females. 67.6% (525) of the deaths identified congenital CMV as an underlying cause and 32.4% (252) identified it as a contributing cause. Neonates (age <28 days) represented 41.1% (319 deaths) of all congenital CMV deaths. Postneonates (age 28–364 days) accounted for 30.6% (238 deaths) of all congenital CMV deaths. Infants (age <1 year), which include both neonates and postneonates, represented 71.7% (557) of all congenital CMV deaths. Children (age ≤5 years) accounted for 83.3% (647) of all congenital CMV deaths. The final 16.7% (130) congenital CMV deaths occurred in those age >5. The 557 congenital CMV-associated deaths in infants during the period 1990–2006 represents 0.11% of the 514,930 total deaths among US infants.

The overall crude mortality rate associated with congenital CMV was 0.17 per 1 million persons annually (95% CI, 0.15–0.18), and after adjustment for age, changed slightly to 0.16 per 1 million persons annually (95% CI, 0.15–0.17) [TABLE 1]. The age-adjusted years of potential life lost for all congenital CMV deaths was 56,355 years. The infant mortality rate associated with congenital CMV was 8.34 per 1 million infants annually (95% CI, 7.65–9.04).

**Table 1:** Congenital CMV-related deaths by sex, race, and region with corresponding age-adjusted mortality rates in the United States, 1990–2006.

Variable	Congenital CMV related deaths, n (%)	Age adjusted mortality rate per 1 million person years, rate (95% CI)
All	777 (100%)	0.16 (0.15, 0.17)
Sex		
Male	340 (43.8%)	0.15 (0.13, 0.16)
Female	437 (56.2%)	0.18 (0.16, 0.19)
Race		
Asian	15 (1.9%)	0.08 (0.04, 0.12)
African American	199 (25.6%)	0.27 (0.23, 0.31)
Hispanic	123 (15.8%)	0.14 (0.11, 0.16)
Native American	15 (1.9%)	0.34 (0.17, 0.51)
White	425 (54.7%)	0.14 (0.13, 0.16)
Region		
Northeast	82 (10.6%)	0.10 (0.10, 0.12)
West	186 (23.9%)	0.16 (0.14, 0.19)
Midwest	183 (23.6%)	0.17 (0.15, 0.19)
South	326 (42.0%)	0.19 (0.17, 0.21)

The age-adjusted mortality rates stratified by race/ethnicity reveal mortality disparities. Native Americans represented 1.9% (15) of all congenital CMV deaths and had the highest age adjusted mortality rate at 0.34 per 1 million annually (95% CI, 0.17–0.51). African Americans accounted for 25.6% (199) of all congenital CMV deaths with an age adjusted mortality rate of 0.27 per 1 million annually (95% CI, 0.23–0.31). Whites represented 54.7% (425) of all congenital CMV deaths with an age adjusted mortality rates of 0.14 per 1 million annually (95% CI, 0.13–0.16). Hispanics represented 15.8% (123) of all congenital CMV deaths with an age adjusted mortality rate of 0.14 per 1 million annually (95% CI, 0.11–0.16). Asians represented 1.9% (15) of all congenital CMV deaths and had the lowest age adjusted mortality rate at 0.08 per 1 million annually (95% CI, 0.04–0.12).

Adjusted rate ratios were calculated for each racial/ethnic group using whites as the reference [TABLE 2]. Native Americans and African Americans were 2.34 (95% CI, 2.11–2.59) and 1.89 (95% CI, 1.70–2.11) times respectively, more likely to die from congenital CMV than whites. Asians and Hispanics were 0.54 (95% CI, 0.44–0.66) and 0.96 (95% CI, 0.83–1.10) times respectively, less likely to die from congenital CMV than whites.

**Table 2:** Frequencies and rate-ratios of congenital CMV-related death by race in the United States, 1990–2006.

Race	Total Deaths	Rate Ratios (whites reference group)	Lower 95% Confidence Interval	Upper 95% Confidence Interval
Asian	15 (1.9%)	0.54	0.44	0.66
African American	199 (25.6%)	1.89	1.70	2.11
Hispanic	123 (15.8%)	0.96	0.83	1.10
Native American	15 (1.9%)	2.34	2.11	2.59
White	425 (54.7%)	referent		

Congenital CMV-associated mortality varied across the 4 major geographic regions of the United States. From 1990–2006, the lowest mortality was found in the Northeast, which had an age adjusted mortality rate of 0.10 deaths per 1 million persons annually. The West, Midwest and South had age adjusted mortality rates of 0.16, 0.17 and 0.19 deaths per 1 million persons annually respectively.

The annual number of deaths from congenital CMV ranged from 36 to 56 deaths with no observed trend over the study period. The number of deaths per month from congenital CMV ranged from 58 to 83 with no obvious seasonal trend noted over the study period.

## Discussion

Congenital CMV causes appreciable mortality in the US and demonstrates important racial/ethnic disparities with African Americans and Native Americans experiencing a disproportionate burden of mortality. Almost three out of every four Congenital CMV deaths occurs during infancy. The greatest Congenital CMV mortality is found in the US South, while the US Northeast experiences the lowest mortality.

The observed mortality disparities are in agreement with the literature demonstrating disparities in CMV antibody seroprevalence among pregnant US women. Staras et al. demonstrated that of seronegative women in the US at age fifteen, 38.1% of whites will seroconvert by age forty-four while 87.3% of African Americans and 63.6% of Mexican Americans will seroconvert by age forty-four [30]. The racial/ethnic disparities in congenital CMV-associated mortality also fit within the context of overall socio-economic disparities in US infant and childhood mortality [31], [32]. Baseline racial/ethnic health disparities and access inequalities likely play a further role in driving this congenital CMV-associated mortality disparity.

In the developing world, CMV is acquired early in life where seroprevalence exceeds 90% by adulthood [1]. The improved, yet varied, living conditions of the developed world have lead to the delayed acquisition of CMV in the US population with those of a minority race and/or lower socioeconomic status experiencing a greater burden of primary infection during the childbearing years (15–44) resulting in an overrepresentation of CMV infection amongst their newborns [3], [8]–[10], .

Control of congenital CMV requires expanded surveillance activities and further elucidation of the epidemiology of CMV to promote vaccine development [2], [4], [20]. The Institute of Medicine placed CMV in its highest priority group for vaccine development based on an analysis of the costs of disease and impact on quality adjusted life years [16]. Despite this, it is claimed that CMV vaccine development is under-funded without prospect of an efficacious vaccine in the near future [9], [33], [37]. A vaccine represents the most desirable public health control measure for the prevention of congenital CMV and may offer further reaching benefits given the burden of disease caused by CMV extends beyond those congenitally infected [38].

Several important limitations are associated with the use of multiple-cause-of-death data that require consideration. Although these data are population based and contain large numbers of observations, death certificates may contain errors, which have been attributed to a variety of factors [39], [40]. The interpretation of cause of death as underlying versus contributing on the death certificate has not been rigorously evaluated [41]. Further, the interpretation of position of the causes is difficult, especially given changes to the original coding by NCHS software.

Underreporting of congenital CMV infections may also limit our study. In order to receive a death certificate, criteria for obtaining a birth certificate must first be met. Congenital CMV infected fetuses that do not survive to live birth are not represented in our data. Furthermore, we have identified over 450 non-congenital CMV-associated deaths in infants in our dataset over the study period, which may contain misclassified congenital CMV-associated cases (data not shown).

In the setting of a severely ill infant, it may be difficult to distinguish an incidental congenital CMV infection from congenital CMV disease. Misclassification on the death certificate could occur if a severely ill infant expired and the physician incorrectly believed the incidental infection to represent disease contributing to death. Unfortunately, we do not have the data that would be necessary to address or estimate the magnitude of this specific bias.

Mortality rates may be distorted because of errors in population estimates, particularly for race/ethnicity. Because estimates of the at-risk population factor into the denominator for rate calculations, such errors can lead to biased estimates. In addition, the small numbers of congenital CMV-related deaths in Native Americans and Asians makes interpretation difficult. Finally, although inferential statistics are not designed for use with population-based data, we have used such methods in order to demonstrate that error does exist within our likelihood ratios. We urge caution in the strict interpretation of our values.

Any efforts to measure the burden of congenital CMV mortality would benefit from having maternal data, however such information is not available in the national multiple cause of death files. Linked birth – infant death data for the US are available from National Center for Health Statistics for selected years and contain valuable information including mother's age, access to prenatal care, gestational age and birth weight; however, only underlying cause of death is provided. Nevertheless, such analyses may provide added insights that are not available from mortality data alone.

## Supporting Information

Checklist S1 STROBE Checklist.
(PDF)
